# Manipulating the Metabolism to Improve the Efficacy of CAR T-Cell Immunotherapy

**DOI:** 10.3390/cells10010014

**Published:** 2020-12-24

**Authors:** Marsha Pellegrino, Francesca Del Bufalo, Biagio De Angelis, Concetta Quintarelli, Ignazio Caruana, Emmanuel de Billy

**Affiliations:** 1 Department of Onco-hematology, Cell and Gene Therapy, Bambino Gesù Children’s Hospital–IRCCS, 00146 Rome, Italy; marsha.pellegrino@opbg.net (M.P.); francesca.delbufalo@opbg.net (F.D.B.); biagio.deangelis@opbg.net (B.D.A.); concetta.quintarelli@opbg.net (C.Q.); Caruana_I@ukw.de (I.C.); 2 Department of Clinical Medicine and Surgery, Federico II University of Naples, 81100 Naples, Italy; 3 Department of Paediatric Haematology, Oncology and Stem Cell Transplantation, University Children’s Hospital of Würzburg, 97080 Würzburg, Germany

**Keywords:** cancer, metabolic reprogramming, combined therapy, Chimeric Antigen Receptor T cells, immunotherapy

## Abstract

The adoptive transfer of the chimeric antigen receptor (CAR) expressing T-cells has produced unprecedented successful results in the treatment of B-cell malignancies. However, the use of this technology in other malignancies remains less effective. In the setting of solid neoplasms, CAR T-cell metabolic fitness needs to be optimal to reach the tumor and execute their cytolytic function in an environment often hostile. It is now well established that both tumor and T cell metabolisms play critical roles in controlling the immune response by conditioning the tumor microenvironment and the fate and activity of the T cells. In this review, after a brief description of the tumoral and T cell metabolic reprogramming, we summarize the latest advances and new strategies that have been developed to improve the metabolic fitness and efficacy of CAR T-cell products.

## 1. Introduction

Chimeric Antigen Receptor (CAR) T-cells are T lymphocytes that have been specifically engineered to target malignant cells [1]. CARs are synthetic molecules designed to activate T cells in response to a specific antigen, mimicking T cell activation through the T cell receptor (TCR) and associated costimulatory molecules. CAR constructs have evolved from the first generation, that included only the signaling endo-domain normally derived from the CD3ζ domain of the TCR or from the γ chain of high-affinity IgE Fc receptor (FcϵRI), to second and third CAR generations by adding and combining different co-stimulatory domains with the aim to increase the efficacy and persistence of the CAR T-cells [2]. The therapeutic successes obtained with CAR T-cells, followed by the approval from the American and European medicines regulatory agencies (Food and Drug Administration (FDA) and European Medicines Agency (EMA), respectively) of two CAR T-cell products targeting the CD19 antigen for the treatment of pediatric/young adult B-cell acute lymphoblastic leukemia (Kymriah^®^) and adult large B-cell lymphoma (Yescarta^®^) [3,4], are the results of many years of research mainly based on the understanding of T cell biology and of their interaction with the surrounding environment [5,6].

Emerging evidence indicates that the metabolism is a key factor in driving the immune response by regulating the activity and the fate of the T cells. From their naïve to highly differentiated effector function, T cells undergo metabolic reprogramming [7]. This allows the T cells to fulfill the increase in energy demand and to generate the intermediate metabolites necessary for their clonal activation, proliferation and differentiation [8]. Cancer cells undergo also metabolic reprogramming in order to promote and sustain their high proliferation rate and survival [9,10]. Moreover, the metabolic reprogramming of cancer cells contributes to the recruitment of cells with immunosuppressive activity and depletes the microenvironment of metabolites and nutriments, creating conditions particularly hostile for T cells to perform proper effector functions [11,12].

CAR T-cells are specifically designed to target an antigen on the surface of cells and they need to be metabolically fit to reach the tumor, survive in an immunosuppressive microenvironment and display their cytolytic function [13]. Because CAR T-cells are easily “manipulable”, either by genetic modifications or by combination with different therapeutic agents, many efforts are being made to identify and develop new strategies to improve their activity against tumors.

In this review, after a brief description of metabolic reprogramming of the tumors and T cells, we summarize the latest advances and new strategies that are proposed to improve the metabolic fitness and the anti-tumor activity of CAR T-cells.

## 2. Metabolism: The Energy Engine

In normal conditions, cells primarily utilize glucose as source of energy to produce adenosine triphosphate (ATP) and sustain their metabolic needs [14]. Through glycolysis, cells metabolize the glucose into pyruvate. Two molecules of pyruvate are reduced into two molecules of Acetyl-CoA, which, together with other Acetyl-CoA molecules deriving from the fatty acid cycle (fatty acid oxidation, FAO) enter the tricarboxylic acid cycle (TCA) for ATP production by the mitochondria [14]. On one hand, these pathways provide the majority of reduced co-enzymes that are subsequently oxidized by the electron carbon chain to produce ATP and, on the other hand, generates intermediate metabolites for the different biosynthetic processes, including gluconeogenesis, lipolysis and amino acid synthesis. Coenzymes such as nicotinamide adenosine dinucleotide (NAD+) and flavin adenine dinucleotide (FAD) are reduced in the TCA cycle and transfer electrons through the electron transport chain to the final acceptor molecule, oxygen (oxidative phosphorylation, OXPHOS). Three NADH^+^ and one FADH_2_ are produced by each TCA cycle and yield through the electron transport chain 10 ATP molecules. This mitochondrial oxidative pathway is referred to as aerobic oxidative metabolism which occurs in the presence of optimal oxygen levels. When the mitochondria are damaged or the oxygen concentration is insufficient, cells convert pyruvate to lactate as a strategy to overcome the adverse condition. Interestingly, both cancer cells and activated lymphocytes share a metabolic strategy dictated by a common metabolic demand, the need to prioritize rapid biosynthesis [15]. For this purpose, the cells choose to shift to the “aerobic glycolysis”, also known as Warburg effect [16], in order to generate readily available intermediates for biosynthesis. Moreover, these cells increase glutamine oxidation to provide a-ketoglutarate to keep the TCA cycle going and produce metabolic intermediates for the biosynthesis of diverse macromolecules [17].

## 3. Effector T Cells and Metabolic Reprogramming

When naïve or quiescent T cells meet the antigen, they become activated, proliferate and, depending on the nature of the antigen stimulation and the microenvironment, differentiate in various T cell subsets with highly specialized defense or regulatory functions [17]. After antigen clearance, most of the effector T cells will disappear by apoptosis while some will remain to constitute a subpopulation of memory T cells [18]. During these different stages and depending on effector or regulatory functions, T cells will adapt their metabolic program according to their needs [19]. Naïve and resting T cells rely mainly on the oxidative phosphorylation and fatty acid metabolism to fulfill their basic demand in energy [20]. Antigen-activated T cells switch rapidly to aerobic glycolysis and glutaminolysis metabolic programs to fuel the high energetic and biosynthetic requirements in amino acids, lipids and nucleotides, necessary to promote and sustain cell proliferation/expansion and survival. Effector T cells use the glycolytic and the FAO pathway, while regulatory T cells (Treg) rely more on the FAO and OXPHOS to maintain their specific functions. However, these metabolic shifts observed in optimal in vitro culture condition appear to be slightly different in vivo. Recent findings indicate that CD8^+^ effector T cells in particular, had a less pronounced shift to glycolysis and a greater rate of oxidative metabolism in vivo compared to what was previously observed in the in vitro studies [21]. When the antigen load decreases, memory T cells switch back to oxidative and fatty acid metabolism resembling the naïve and quiescent T cell metabolic phenotype [22]. Under circumstances of chronic immune activation or continuous antigen stimulation, as observed for chronic viral infection and cancer, the activity of effector T cells decreases over time and they eventually undergo apoptosis, impairing formation of memory T cell sub-population. This process, known as exhaustion, is characterized by a metabolic dysfunction associated to a reduction in mitochondrial mass and ATP production [23,24]. Consequently, a decrease in cytokine production, such as interleukin-2 (IL-2), essential for T cell proliferation and function, and an increase in the expression of immune checkpoint inhibitors including programmed cell death protein 1 (PD-1), cytotoxic T-lymphocyte-associated protein 4 (CTLA-4) and T cell immunoglobulin and mucin domain-containing protein 3 (TIM-3), leading to a shutdown of the immune response [25,26].

## 4. Tumor Metabolism and Microenvironment

Tumors are a heterogeneous group of diseases involving abnormal cell growth with the potential to invade and spread throughout the body. This abnormal replication rate requires a continuous source of energy and a modification of the normal metabolism, which induces important modifications of the microenvironment capable of limiting the survival of elements of the immune system. Tumor cells can organize and generate masses which may have different characteristics based on their localization. For example, in the inner part of the tumor mass, the poor vascularization strongly reduces the oxygen levels and, thus, cells cannot rely on normal oxidative metabolism to survive and proliferate [27]. In this hypoxic condition, a decrease in ATP production is observed leading to an increase in glucose and glutamine uptake, which, once converted into pyruvate, is not metabolized into Acetyl-CoA to fuel the oxidative path normally required for maximum ATP production. On the contrary, pyruvate is directed toward the production of lactic acid. This switch in metabolic program from the oxidative to the lactic acid fermentation leads to an increase in production and release of lactic acid by the tumor cells, contributing to the acidification of the tumor microenvironment (TME) [28].

Both these phenomena, the acidification and the hypoxia, are interconnected with each other and promote important molecular changes able to increase the tolerance to the acidosis, facilitating tumor evolution, growth and metastasis but also to increase the resistance to pharmacological intervention [29,30]. In particular, under hypoxic conditions, tumor cells respond with a rapid induction of the transcription factors hypoxia-inducible factor 1 alpha (HIF1α) and Nuclear Factor kappa-light-chain-enhancer of activated B cells (NFKB), both involved in the regulation of genes implicated in inflammation and adaptation to hypoxia [31]. They control the mitochondrial dynamic and mitophagy, promoting the acidification of the microenvironment [32,33,34]. Furthermore, these transcription factors regulate the expression of a plethora of interleukins/cytokines and angiogenic factors [IL-6, IL-10, IL-1β, chemokine (C-X-C motif) ligand 8 (CXCL8), vascular endothelial growth factor (VEGF)] [35,36,37,38]. These molecules help recruitment of cells with immunosuppressive functions, including myeloid-derived suppressor cells (MDSCs), Treg, Innate Lymphoid Cells (predominantly 2 and 3), Tumor-Associated Macrophages and Cancer-Associated Fibroblasts to establish an immunosuppressive/tolerogenic environment [39] and, suppress antigen processing and presentation favoring immune evasion [40,41].

HIFα and NFKB control also the expression of the checkpoint molecule programmed death-ligand 1 (PD-L1), which binds to the checkpoint inhibitor PD1 receptor present at the surface of antigen-activated T cells, inhibiting their cytolytic activities [42,43,44]. The PD-L1/PD1 axis is critical in the regulation of T cell metabolism. PD1 blocks glycolysis, through the inhibition of the phosphoinositide 3-kinase (PI3K)/protein kinase B (PKB)/mammalian target of rapamycin (mTOR) pathway and the down-regulation of glucose transporter 1 (GLUT1) expression, both essential for T cell activation [45]. Moreover, PD1 activates 5′-prime-AMP-activated protein kinase (AMPK), a kinase involved in the regulation of the FAO, and induces autophagy through activation of the Unc-51 like autophagy activating kinase (ULK1) [46]. On the other side, PD-L1 drives glycolysis in tumor cells and contributes to the depletion of the glucose from the TME [47], an essential element for CAR T-cell activity. In addition to the PD-L1/PD-1 axis, other immune checkpoint molecules, including CTLA-4, lymphocyte activation gene 3 (LAG-3), TIM-3, B and T lymphocyte attenuator (BTLA), T cell immunoreceptor with Ig and ITIM domains (TIGIT) or V-domain Ig suppressor of T cell activation (VISTA), engage with specific molecules expressed at the surface of the tumor cells and inhibit T cell proliferation, cytokine production and cytolytic function [48]. However, their function and mechanism of actions in the regulation of T cell metabolism are not completely understood. TIM-3 has recently been linked to the regulation of T cell glucose metabolism [49] by down-regulating glucose uptake and consumption, as well as by increasing the release of lactate. LAG-3 maintains mitochondrial and metabolic quiescence in naïve CD4^+^ T cells potentially through the regulation of the signal transducer and activator of transcription 5 (STAT5) pathway [50]. CTLA-4 may have a role in the catabolism of tryptophan in immunosuppressive metabolite [51], while TIGIT regulates glucose uptake and impair T cell effector function [52].

The activation of HIF1α leads also to the up-regulation of the expression of CD39 and CD73, enzymes involved in the conversion of the extracellular ATP in adenosine [53] which affects the function and activity of several cell types of the immune system through its binding to the G-coupled receptor adenosine 2A receptor (A_2A_R). Adenosine increases the activity of the immunosuppressive cells, such as Tregs, while inhibits the recruitment, infiltration and the activation of effector T cells and natural killer (NK) cells [54].

**Figure 1:**
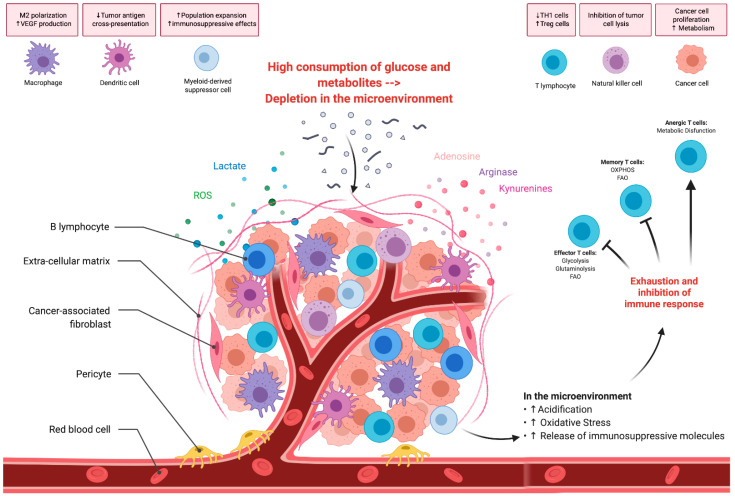
Tumor cell metabolism and immunosuppression. The figure shows the different ways by which the tumor cell metabolic reprogramming conditions the tumor microenvironment and affects the immune response. Treg, regulatory T cells; OXPHOS, oxidative phosphorylation; FAO, fatty acid oxidation; VEGF, vascular endothelial growth factor; ROS, reactive oxygen species, Th1, helper T cells. Created with Biorender.

Depletion of the tryptophan from the TME and its conversion by malignant cells in kynurenines (quinolinic and 3-hydroxyanthranilic acids), by the indoleamine 2,3-dioxygenase enzyme, is another important element involved in the regulation of the tumor immunity [55,56,57]. Once released in the TME, kynurenines facilitate tumor progression and metastasis, induce T cell differentiation into regulatory T cells and suppress helper and effector response by inducing apoptosis [58,59] (Figure 1).

## 5. Armoring CAR T-cells: Improving the Intrinsic Anti-Tumor Activity through Improved Metabolic Fitness

CAR T-cells, like other effector T cells, require specific metabolic support for optimal performance in terms of proliferation and maintenance of their specific effector and memory functions. Since the therapeutic response of CAR T-cell treatment in patients is strictly linked to their activities and persistence, many efforts have been committed to maximize CAR T-cell efficacy and rendering cells metabolically fit to deal with the tumor.

### 5.1. Engineering of the CAR Module: Costimulation as Metabolic Support for T Cells

Second and third generation of CARs are composed of a combination of costimulatory domains such as immunoglobulin (Ig) superfamily members, CD28 or inducible T cell costimulatory (ICOS), and the tumor necrosis factor receptor (TNFR) superfamily members 4-1BB, OX40 and CD27. Depending on the costimulatory domains incorporated into the synthetic CAR construct, different signaling pathways are triggered upon antigen activation [1,60,61]. These co-stimulatory domains are particularly implicated in the regulation of T cell metabolic reprogramming, mimicking a physiologic response and improving their persistence, memory and anti-tumor potency.

Antigen activation of second-generation CAR integrating a CD28 cytoplasmic domain (CD28.CD3ζ) enhances the glucose uptake and the aerobic glycolysis, which correlates with an increase in the effector T cell memory population [62]. Glycolysis induction observed after CD28 stimulation appears to be promoted through the activation of PKB/mTOR signaling pathway and activation of HIF1α, the latter being directly involved in the up-regulation of glucose uptake and the expression of glycolytic enzymes [63]. However, other evidence demonstrates that the tonic activation of CAR T-cells with the CD28 endo-domains is responsible for the suboptimal anti-tumor activity observed in vivo as the T cells exhaust rapidly, resulting into a decrease in cell proliferation and cytokine production [63].

In comparison, T cells transduced with second-generation CAR constructs comprising the 4-1BB domain (4-1BB.CD3ζ) have an enhanced mitochondrial biogenesis and oxidative metabolism, which is associated with an increase in cell survival and central memory T cell population. Activation of a 4-1BB.CD3ζ CAR construct targeting CD19 was also reported to counteract the effect of chronic CAR signaling stimulation by decreasing exhaustion and increasing central memory-related markers as well as by inducing a gene expression signature related to hypoxia, metabolism and apoptosis [64]. Activation of 4-1BB increases the metabolic capacity of the T cells through a peroxisome proliferator-activated receptor gamma coactivator 1-alpha (PGC1α)-dependent mitochondrial fusion and biogenesis mechanisms, via the activation of the p38-microtubule associated protein kinase (MAPK) pathway [63,65].

Therefore, 4-1BB induces a higher mitochondrial oxidative phosphorylation upon activation allowing the generation of memory T cells with a better in vivo persistence phenotype, while CD28 activation increases the aerobic glycolysis path leading to an early dominance of the effector T cells. These differences in T cell phenotypes associated with their metabolic programs are in agreement with clinical observations showing that, T cells transduced with CD19.CAR-4-1BB.CD3ζ construct demonstrate superior efficacy in acute lymphoblastic leukemia than those transduced with the CD19.CAR-CD28.CD3ζ construct [66]. CD28 and 4-1BB endo-domains regulating the effector and the memory T cell phenotypes, respectively, appear both critical for CAR T-cell activity and third-generation CAR built with a combination of these two co-stimulatory domains (CD28.4-1BB.CD3ζ) demonstrated superior anti-tumor efficacy in vitro and in vivo preclinical models compared to their respective second generation CARs (CD28, OX40, 4-1BB) and a third generation CAR encoding for CD28.OX40 costimulatory domains [67]. While CD28 and 4-1BB cytoplasmic domains appear both critical for CAR T-cell activity, other co-stimulatory domains are reported to regulate the metabolism and to potentiate the anti-tumor activity of the CAR T-cells. For instance, dual costimulation of 4-1BB and OX40 in melanoma enhanced glucose uptake, glycolysis, and OXPHOS [68]. OX40, normally induced after T cell activation, regulates Tregs glycolysis and lipid metabolism and promotes T cell expansion and generation of memory cells through a TNF receptor-associated factor 2 (TRAF2)-dependent mechanism [69]. However, Quintarelli et al. demonstrated that OX40 incorporated into third-generation CARs, CD28.OX40.CD3ζ or 4-1BB.OX40.CD3ζ decreases INFγ and IL2 production and the anti-tumor activity when compared to a CAR construct including the combination of CD28.4-1BB.CD3ζ [67].

Another member of the TNFR superfamily costimulatory proteins is CD27, which is normally expressed in resting T cells [70]. Integration of the CD27 cytoplasmic domain into a CAR construct enhances T cell expansion, effector functions as well as survival and augments T cell persistence and anti-tumor activity in vivo. These effects are potentially mediated through the induction of anti-apoptotic proteins of the B-cell lymphoma 2 (Bcl-2) family and the up-regulation of the proto-oncogene serine/threonine-protein kinase Pim-1, particularly involved in the regulation of the oxidative stress and aerobic glycolysis [71,72].

Stimulation of ICOS, a member of the immunoglobulin superfamily costimulatory molecules, switch on the glycolysis and lipogenesis pathways through activation of mTORC1 and mTORC2, as well as the induction of Glut-1 and is a key player for the differentiation and expansion of helper T cells (Th17) [73]. CARs with ICOS cytoplasmic domain are linked to immunotherapies that require Th17 cell function and prevalence [74]. However, second-generation ICOS-based CAR showed to increase the anti-tumor activity and persistence of the transduced T cells when compared to CARs with CD28 and 4-1BB intracellular-domains [75]. Moreover, CAR T-cell persistence and anti-tumor activity were further enhanced when ICOS was combined with the 4-1BB in a third-generation CAR.

All these observations indicate clearly that depending on the co-stimulatory domains integrated in the CAR construct, T cells will activate different metabolic pathways with a particular impact on their functions, fitness and behavior inside the TME.

### 5.2. Exploiting Transcription Factors and Specific Genes Pathways to Promote Potent Antitumor Activity of CAR T-Cells

Transcription factors regulate the expression of a specific set of genes, some of which are particularly involved in modifying the metabolic states of the T cells. Therefore, different strategies to modulate their activity and modify CAR T-cells transcriptional programs have been used to increase the metabolic fitness and intrinsic anti-tumor activity of engineered T cells.

Kagoya et al. have modified a CAR construct to activate specifically STAT3 [76], a transcription factor involved in the rapid innate immune mitochondria reprogramming and inflammatory response upon antigen stimulation [77]. The modified CAR construct contained a truncated domain of the Il2 receptor and a STAT3 binding tyrosine motif (YXXQ), as well as the co-stimulatory domain CD28. Upon stimulation, STAT3 pathway is activated and the CAR T-cells show potent cytotoxic activity even after repetitive antigen stimulation in vitro resulting in a superior anti-tumor effect in vivo when compared to T cells transduced with a CD28 or 4-1BB second-generation CAR. Over-expression of IL23 is another way to induce STAT3 activation in CAR T-cells. IL23 is constituted by two sub-units, IL23aP19 and IL12bp40, which only assemble upon T cell activation. Xingcong Ma et al. have co-expressed the CAR construct with the P40 subunit, which binds the P19 sub-unit to form IL23 only upon antigen activation [78]. As a result, these IL23-engineered CAR T-cells increase their proliferation rate and lytic activity, as well as decrease the expression of exhaustion markers in vitro. In vivo, the CAR T-cells demonstrate a better tumor control and improve survival. Another approach used to activate the STAT pathway in CAR T cells was to co-expressed together with the CAR a membrane-bound chimeric IL15. The engineered T cells signal through the STAT5 pathway, maintaining a memory-like transcriptional profile and developing a long-term persistence phenotype in vivo [79].

More recently, Kondo et al. have shown that the activation of the Notch homolog 1 facilitates mitochondrial biogenesis, fatty acid synthesis and OXPHOS in CAR T-cells targeting CD19 and leads to the maintenance of a stem cell-memory T cell phenotype [80]. They further demonstrated that the NOTCH effect is mediated through the induction of the transcription factor forkhead box M1 (FOXM1) and the metabolic reprogramming of the T cells. Moreover, overexpression of FOXM1 in CAR T-cells enhances their anti-tumor activity and their stem cell-memory phenotype in an in vivo model of leukemia.

The CAR T-cell metabolic program can also be improved using modified transcription factors as exemplified with the T-box transcription factor TBX21 (T-Bet). T-Bet is normally highly expressed in T cells and is required for the regulation of genes involved in the proinflammatory pathway and the development of T helper (Th) CD4^+^ cells into a Th1 phenotype [81]. The co-expression of T-bet with a second-generation CAR (CD28.CD3ζ) targeting the B7-H3 antigen has been shown to potentiate CAR T-cell anti-tumor activity. This effect was observed with a T-bet construct deleted in its DNA binding domain (ΔTBOX), indicating that it is unlikely mediated through its direct transcriptional activity. The use of the ΔTBOX construct is known to upregulate the expression of glycolytic pathway genes through the binding of its transactivation domain with other transcription factors, such as BCL6 or NFKB [82,83].

Modifications of the transcription factor activities have been also performed using specific small molecules. The down-regulation of the basic leucine zipper ATF-like transcription factor (BTAF) expression, which cooperates with interferon regulatory factor 4 (IRF4) and nuclear factor of activated T cells (NFAT) to impair CD8^+^ T cell metabolism and promote exhaustion, has been achieved using JQ1, an inhibitor of the bromodomain containing protein 4 (BRD4) epigenetic regulator [84]. The decrease in BTAF expression following JQ1 treatment led to an increase in glycolysis and OXPHOS, which maintain CD8^+^ T cells with features of stem cell-like and central memory phenotypes, and co-administration of JQ1 with CAR T-cells enhanced their anti-tumor activity and persistence in vivo. C-Jun over-expression in CAR T-cells has been proposed to be another strategy to negatively regulate the BTAF involvement in T cell exhaustion. C-jun appears to compete with BTAF/IRF at the promoter of the genes switching off BTAF transcriptional activities. Over-expression of C-jun protects CAR T-cells from exhaustion by enhancing IL2 expression and inhibiting the transcription activity of the BTAF/IRF [85].

While the different strategies to manipulate the activity of specific transcription factors have been shown to potentiate CAR T-cells by modifying their metabolism, the efficacy and the safety of these engineered T cells remains to be demonstrated in the clinical arena.

## 6. Pre-Conditioning CAR T-Cells to Increase Their Metabolic Fitness

The improvement of the quality and fitness of the CAR T-cells before their adoptive transfer is an important step. The composition of the culture medium and the protocol for the expansion of CAR T-cells are crucial to tune specific metabolic programs and enhance CAR T-cell persistence and/or cytotoxic activity and therefore their anti-tumor activity.

The addition of specific cytokines in the culture medium, such as IL7 and IL15, have been carefully chosen to enhance the fitness of the CAR T-cells [86]. IL7 enhances glucose uptake through the STAT5 activation pathway, increasing the survival of the CAR T-cells [87]. On the other hand, IL15 reduces mTOR activity and the expression of glycolytic enzymes, but improves mitochondrial fitness, favoring the stem cell-like properties of CAR T-cells [88]. The inhibition of mTOR activity in IL2 stimulated CAR T-cells with rapamycin or with dichloroacetic acid, both known to block aerobic glycolysis, has shown similar results to IL15 treatment on the CAR T-cell differentiation but impairs their expansion ex-vivo. However, incubation of CAR T-cells with inhibitors of PI3K, upstream regulator of mTOR, increase the naïve and central memory T cell sub-population without affecting ex-vivo expansion. Moreover, PI3K and PKB inhibitors enhance CAR T-cells in vivo persistence and anti-tumor activity [89,90].

The nutritional component of the culture medium also needs to be optimized in order to improve adoptive transfer of T cell therapy. For example, Geiger et al. observed that increasing L-arginine levels lead to phenotypic changes in TCR transgenic CD8^+^ OT-I T cells [91]. L-arginine enhances T cell metabolic fitness by increasing OXPHOS and decreasing glycolysis and, therefore, induces a central memory-like phenotype that improved persistence and anti-tumor activity in vivo. Thus, CAR T-cells could be pre-incubated with specific metabolites such as L-arginine before their adoptive transfer to the patient. Another strategy, recently highlighted by the work of Fulthan et al. [92], demonstrates that CAR T-cells are susceptible to low arginine level because of the low expression of the resynthesis enzymes, ornithine transcarbamylase and argininosuccinate synthase. Co-expressing these enzymes in a 4-1BB second-generation CAR showed a metabolic rewiring toward arginine and proline, as well as pyrimidine and purine metabolisms. As a result, the proliferation of the modified CAR T-cells is enhanced in vitro and the antitumor efficacy is significantly improved for different in vivo pre-clinical tumor models [92].

Inhibition of the lymphocyte cell-specific protein-tyrosine kinase (LCK) with dasatinib is another way to condition CAR T-cell activity. Dasatinib inhibits LCK-induced CD3ζ phosphorylation of the CAR construct and, thus, blocks CAR T-cell activation, proliferation, cytokine production and anti-tumor activity in vivo without affecting their viability. This blockade is rapidly and completely reversible following removal of dasatinib. Therefore, dasatinib can be used as a pharmacologic on/off switch to control CAR T-cell activity and associated toxicity, such as cytokine release syndrome [93].

## 7. Molding the Tumor Microenvironment: Improving Extrinsic Factors to Contrast Adverse TME

### 7.1. Adapting the Strategy to the Specific Situation

To cope with hypoxia, where the expansion, differentiation and cytokine production of CAR T-cells is impaired [94], Jiullerat et al. [95] have proposed a strategy where the expression of the CAR is stabilized and therefore functional only in hypoxic condition. This was performed by adding a particular subdomain of the transcription factor HIF1α to the CAR module, which normally regulates the degradation or the stabilization of HIF1α protein depending on the level of oxygen [95]. While these engineered CAR T-cells did not significantly improve their cytotoxic activity in vitro, the author proposes that the integration of the tumor microenvironment sensor may minimize the on-target/off-tumor effect and expand the number of antigens surface for therapeutical purpose.

In addition to the acidification of the TME, hypoxia increases the production of H_2_O_2_ creating oxidative stress and generating reactive oxygen species (ROS) that help tumor cell growth. In addition, the mitochondrial ROS production is increased in antigen-activated CAR T-cells and appears to play a crucial role in activation, differentiation and metabolic reprogramming of the cells [96]. However, uncontrolled ROS production provokes systemic T cell dysfunction and abrogation of cytokines production [97]. To counteract the high level of ROS, Ligtenberg et al. have over-expressed catalase in CAR T-cells, this enzyme being capable of metabolizing H_2_O_2_ [98]. The CAR T-cells over-expressing catalase demonstrate less oxidative stress upon activation and a superior ability to explicit their cytotoxic function under high concentration of hydrogen peroxide in vitro. Moreover, they were also able to protect bystander T and NK cells from oxidative stress-dependent inhibition. Although this approach may present several advantages in vitro, its relevance for anti-tumor therapy remains to be demonstrated in vivo. 

The tumor growth factor beta (TGFβ) is a cytokine that can be secreted by tumor cells and is particularly abundant in the TME [99]. In addition to promoting tumor growth and contributing to lactate production [100,101], TGFβ has a clear function in immunosuppression and tumor cell evasion by modifying the fate of T cells. TGFβ induces the differentiation of CD4^+^ T cells into immunosuppressive Treg cells by inhibiting glycolysis and maintaining the expression of forkhead box P3 (FOXP3) [102]. Moreover, it suppresses the cytotoxic activity of CD8^+^ T cells [103]. In CAR T-cells, TGFβ induces also a Treg-like phenotype and accelerates their exhaustion through the activation of the transforming growth factor beta receptor II (TGFBR2). Knockdown, using CRISP/Cas9 technology, or over-expression of a dominant-negative TGFB2R, render the CAR-T cells resistant to exhaustion and considerably improve their anti-tumor activity and persistence. TGFβ-resistant CAR T-cells are now being tested in ongoing clinical trials (NCT03089203, NCT00889954, NCT02065362) [104,105].

Recently, several studies highlighted the role of mitochondria in regulating key processes in T lymphocytes. It has long been recognized that memory T cells mainly rely on mitochondria-based oxidative metabolism [106,107]. Interestingly, the morphology of the mitochondrial network is tightly linked to the cell metabolic status and it can be actively controlled. As demonstrated by Buck et al. [108], pharmacological manipulation using a mitochondrial “fusion promoter” compound was able to favor mitochondria elongation and OXPHOS activity and to reprogram T cells towards a memory phenotype favoring their long-term survival and increasing their anti-tumor function. As it is well known that mitochondria also regulate T cell migration, proliferation and apoptosis, all key aspects necessary to an optimal anti-tumor response, modulation of their dynamics may, therefore, represent an important strategy to increase T cell fitness, invasiveness and expansion [109,110].

### 7.2. Checkpoint Blockade and CAR T-Cell Therapy

As for T cells, CAR T-cells, after their activation, have been found to express on their cell surface inhibitory molecules, including PD1, CTLA4, LAG-3, TIM3 and A_2A_R. Theseobservations, made in vitro and in clinic with T cells expressing different CAR constructs, led to the development of several strategies to counteract the inhibitory effect of these molecules and to revert the exhausted phenotype of the CAR T-cells [111].

As mentioned earlier, the PD-L1/PD1 axis has a clear impact on both tumor- and T cell metabolism. Blockade of PDL-1 decreases the tumor glycolytic pathway through the inhibition of the PI3K/mTOR pathway and glucose uptake, leading to an increase availability in glucose in the TME required for the activation of CAR T-cells. On the other side, PD1 acts primarily by inhibiting the CD28 subdomain integrated in CAR constructs, and its blockade reverts the metabolic shutdown and rescues the exhausted T cell phenotype. Blocking the PD-L1/PD1 axis either by using neutralizing monoclonal antibody such as anti-PD1 (pembrolizumab, nivolumab, cemiplimab), anti-PDL1 (atezolizumab, durvalumab, avelumab) [112] or by engineering CAR T-cells to secrete one or a combination of antibodies has been shown to boost CAR T-cell efficacy [113,114,115]. Other strategies tested the overexpression of a PD1 dominant negative receptor or a chimeric PD1:CD28 construct [116,117] as well as inhibiting its expression using the short hairpin RNA (shRNA) or the gene editing CRISPR/Cas9 technologies [118]. All of these approaches have been shown to potentiate the anti-tumor activity and to increase the persistence of CAR T-cells. While several clinical trials are ongoing to explore the efficacy of these approaches in patients affected by different lymphoid and solid tumor types [119], the effectiveness of PD1/PDL-1 blockade in combination with CAR T-cells is not always as expected. For examples, PD1 inhibition in combination with disialoganglioside (GD2).CAR T-cells did not demonstrate a significant effect in relapsed or refractory neuroblastoma patients infused with GD2.CAR T-cells [120]. To improve efficacy, combined inhibition of PD1/PDl-1 pathway together with other checkpoint molecules are now being tested [121,122,123,124]. For instance, the efficacy and safety of CAR T-cells co-expressing CTLA4 and PD-1 antibodies is now evaluated in clinical trials on epidermal growth factor receptor (EGFR) positive solid tumors (NCT03182816, NCT03182803).

**Figure 2:**
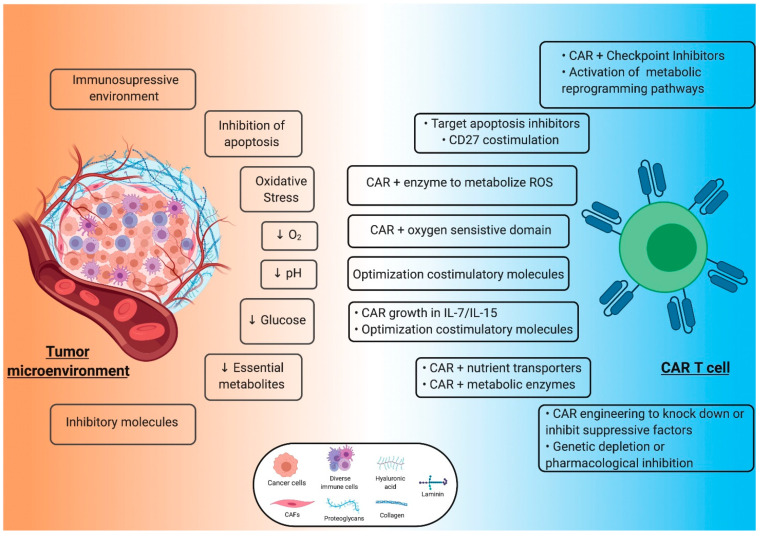
Tumor microenvironment and chimeric antigen receptor (CAR) T-cell immunotherapy strategies. The figure shows the different strategies developed to improve CAR T-cells metabolic fitness and anti-tumor activity in response to the hostile immunosuppressive effect of the tumor metabolism. CAFs, cancer associated fibroblasts. Created with Biorender.

The conversion of ATP in adenosine by tumor cells suppresses CAR T-cell activity and mobility [125]. Genetic depletion or the pharmacologic inhibition with adenosine analogues (SCH58261) of the A_2A_R expressed by activated CAR T-cells, enhance their anti-tumor function. This effect was further boosted when the A_2A_R antagonist were used in combination with PD-1 blockade in in vivo models of receptor tyrosine-protein kinase erbB-2 (HER2) positive breast tumors [126]. The production by the tumor cells of tryptophan metabolites, such as Kynurenines, also has a potent immunosuppressor effect on CAR T-cells. Kynurenines inhibit proliferation, anti-tumor activity, production of IL2 and INFγ and induce CAR T-cell apoptosis. Inhibition of the enzyme responsible of the tryptophan catabolism, indoleamine-pyrrole 2,3-dioxygenase (IDO) 1, with 1-methyl-tryptophan restored CAR T-cell activity against IDO1 positive tumors in vivo in a preclinical study [127] (Figure 2).

## 8. Conclusions

The discovery and implementation of various strategies have led to a tremendous improvement of CAR T-cell therapeutic efficacy and safety, with, however, limited success for solid tumors. The efficacy of CAR T-cell treatment and the patient survival depend on how efficiently CAR T-cells perform, this being correlated to and influenced by the metabolic fitness of the engineered T cells, as well as to the metabolic state of the tumor and the composition of its microenvironment. Harnessing the tumor metabolism to impede its immunosuppressive effect while improving the metabolic abilities of the CAR T-cells appears a promising approach to optimize the efficacy of this promising immunotherapy. However, the high heterogeneity and complexity of the tumors and microenvironment may imply the engineering of CAR T-cells specifically designed, or their use in combination with selected agents, depending on the need. This will necessarily require a better understanding of the immunomodulatory landscape of the TME on T lymphocytes using diverse metabolomic, proteomic and transcriptomic technologies, as well as data integration for the identification of specific biomarkers and the design of more robust and effective CAR T-cell therapeutic strategies. Furthermore, these approaches need to be validated in pre-clinical studies including both long- and short- toxicity tests considering the characteristic, source and strategy of production of the effector cells used for the generation of CAR^+^ cells (polyclonal T cells, antigen-specific T cells, CD8^+^, a particular ratio of CD4^+^/CD8^+^, γ/δ T cells, NK-T cells or NK cells), for which, however, only limited data are now available on their metabolic response to the in vitro stimulation and their anti-tumor response.

## Abbreviations

CAR, chimeric antigen receptor; FDA, Food and Drug Administration; EMA, European Medicines Agency; ATP, adenosine triphosphate; FAO, fatty acid oxidation; TCA, tricarboxylic acid cycle; NAD, nicotinamide adenosine dinucleotide; FAD, flavin adenine dinucleotide; OXPHOS, oxidative phosphorylation; Treg, regulatory T cells; IL, Interleukin; PD-1, programmed cell death protein 1; CTLA-4, cytotoxic T-lymphocyte-associated protein 4; TIM3, T cell immunoglobulin and mucin domain-containing protein 3; TME, tumor microenvironment; HIF1α, hypoxia-inducible factor 1 alpha; NF-kB, Nuclear Factor kappa-light-chain-enhancer of activated B cells; CXCL, chemokine (C-X-C motif) ligand; VEGF; vascular endothelial growth factor; MDSC; myeloid-derived suppressor cells; PD-L1, programmed death-ligand 1; PI3K, phosphoinositide 3-kinase; PKB, protein kinase B; mTOR, mammalian target of rapamycin; GLUT1, glucose transporter 1; AMPK, 5′-prime-AMP-activated protein kinase; ULK1, Unc-51 like autophagy activating kinase; LAG3, lymphocyte activation gene 3; BTLA, B and T lymphocyte attenuator; TIGIT, T cell immunoreceptor with Ig and ITIM domains; VISTA, V-domain Ig suppressor of T cell activation; STAT5, signal transducer and activator of transcription 5; A_2A_R, adenosine 2A receptor; NK, natural killer; TCR, T cell receptor; FcϵRI, IgE Fc receptor; Ig, immunoglobulin; ICOS, inducible T cell costimulatory; TNFR, tumor necrosis factor receptor; PGC-1α, peroxisome proliferator-activated receptor gamma coactivator 1-alpha; MAPK, microtubule associated protein kinase; TRAF2, TNF receptor-associated factor 2; INFγ, interferon gamma; Bcl-2, B-cell lymphoma 2; Pim-1, Proto-oncogene serine/threonine-protein kinase; Th17, helper T cells; STAT3, signal transducer and activator of transcription 3; NOTCH1, Notch homolog 1, translocation-associated; FOXM1, forkhead box M1; T-bet, T-box transcription factor TBX21; BTAF, Basic leucine zipper transcription factor, ATF-like; IRF4, interferon regulatory factor 4; NFAT, nuclear factor of activated T cells; BRD4, bromodomain containing protein 4; LCK, lymphocyte cell-specific protein-tyrosine kinase; Th, T helper; ROS, reactive oxugen species; TGFβ, tumor growth factor beta; FOXP3, forkhead box P3; TGFBR2, transforming growth factor, beta receptor II; shRNA, short or small hairpin RNA; GD2, disialoganglioside; EGFR, epidermal growth factor receptor; HER2, receptor tyrosine-protein kinase erbB-2; IDO, indoleamine-pyrrole 2,3-dioxygenase; CAFs, cancer associated fibroblasts.
